# Toward the Assembly of 2D Tunable Crystal Patterns of Spherical Colloids on a Wafer-Scale

**DOI:** 10.1021/acsami.3c16830

**Published:** 2024-01-25

**Authors:** Kai Sotthewes, Gijs Roozendaal, Andris Šutka, Ignaas S. M. Jimidar

**Affiliations:** † Physics of Interfaces and Nanomaterials, MESA+ Institute, University of Twente, P.O. Box 217, 7500AE Enschede, The Netherlands; ‡ Mesoscale Chemical Systems, MESA+ Institute, University of Twente, P.O. Box 217, 7500AE Enschede, The Netherlands; § Institute of Materials and Surface Engineering, Faculty of Materials Science and Applied Chemistry, Riga Technical University, LV-1048 Riga, Latvia; ∥ Department of Chemical Engineering CHIS, Vrije Universiteit Brussel, Brussels 1050, Belgium

**Keywords:** dry particle assembly, crystals, ordered arrays, colloidal particles, tribocharging

## Abstract

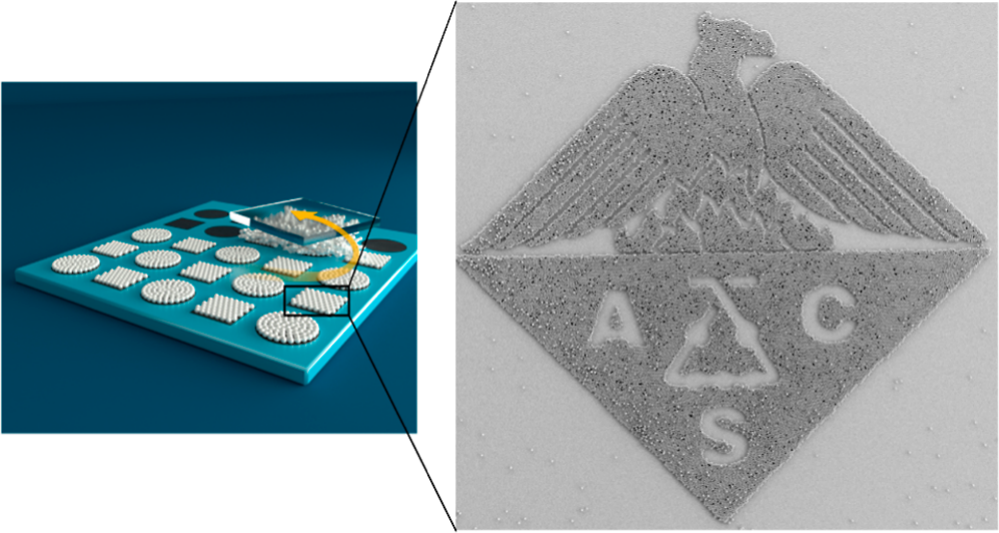

Entering an era of miniaturization prompted scientists to explore strategies to assemble colloidal crystals for numerous applications, including photonics. However, wet methods are intrinsically less versatile than dry methods, whereas the manual rubbing method of dry powders has been demonstrated only on sticky elastomeric layers, hindering particle transfer in printing applications and applicability in analytical screening. To address this clear impetus of broad applicability, we explore here the assembly on nonelastomeric, rigid substrates by utilizing the manual rubbing method to rapidly (≈20 s) attain monolayers comprising hexagonal closely packed (HCP) crystals of monodisperse dry powder spherical particles with a diameter ranging from 500 nm to 10 μm using a PDMS stamp. Our findings elucidate that the tribocharging-induced electrostatic attraction, particularly on relatively stiff substrates, and contact mechanics force between particles and substrates are critical contributors to attain large-scale HCP structures on conductive and insulating substrates. The best performance was obtained with polystyrene and PMMA powder, while silica was assembled only in HCP structures on fluorocarbon-coated substrates under zero-humidity conditions. Finally, we successfully demonstrated the assembly of tunable crystal patterns on a wafer-scale with great control on fluorocarbon-coated wafers, which is promising in microelectronics, bead-based assays, sensing, and anticounterfeiting applications.

## 1 Introduction

Crystal structure formation comprising micro- and nanoparticles has been the backbone for a plethora of studies as these crystals serve as a suitable candidate for comprehending phase transformations from a fundamental perspective,^1,2^ while they have also proven to be promising for vast applications, e.g., photonics, colloidal lithography, liquid chromatography, optical sensors, sensing, strain sensors, antireflective surfaces, self-cleaning surfaces, and conductive films.^3−11^ The challenging game scientists play in balancing the rich complexity of surface interaction forces^3,12^ to assemble particles in an ordered structure shapes the landscape of various assembly methods proposed over the last decades.

Broadly taken, we can distinguish between wet and dry assembly methods. A few wet assembly techniques include dip-coating, convective assembly, spin-coating, wet rubbing assembly, and electrostatic/electrophoretic deposition.^3,10,13,14^ However, most wet techniques suffer from leaving residues and large scalability during manufacturing as they hinge on balancing solvent properties, optimized evaporation rate, particle size, and material properties. Despite this, a formidable quantity of wet assembly methods are being proposed, while the development and physical understanding of dry assembly techniques is lacking. The latter is surprising as dry assembly methods can be faster and more applicable to a larger spectrum of particle types. So far, proposed dry assembly methods include mechanical rubbing^15−17^ or agitation,^14,18^ while more recently, vacuum-driven assembly with the aid of electrostatic levitation^19^ or powder impact^20^ has been explored. These studies reported the use of hard (silicon) or soft (polydimethylsiloxane (PDMS)) physically templated surfaces, i.e., surfaces covered with periodic holes or wells to attain ordered, nonclosely packed particle arrays. The fabrication of these physically templated substrates renders the assembly process inevitably more time-consuming and costly.

On the other hand, the manual rubbing method was previously also explored using bare fingertips or small rubber pieces to attain closely packed colloidal crystals,^21−23^ but the ordering of particles into crystalline structures was rather poor. In these studies, the assembly of dry powder particles on substrates was driven by the concept of hydrogen bonding between the particles and substrates, which causes particles to adhere to the substrate.^24^

The Jeong group markedly improved the rubbing method by utilizing soft elastomeric substrates with a Young’s modulus of ≈3 MPa, e.g., PDMS, to cover large areas with single crystals.^25,26^ In a follow-up study, they successfully obtained patterned arrays of colloidal particles on flexible physical templated substrates.^16^ As they utilized elastomeric substrates with a relatively low Young’s modulus, the manual rubbing assembly of the particles is based on creating sufficient adhesion between the particles and the elastomeric substrates by means of the dominant JKR contact model,^27^ i.e., emphasis was put on contact mechanics force-driven assembly. However, the Jeong group’s work has been limited to utilize particles between two PDMS sheets (both rubbing stamp and substrate) and other types of similar rubbery substrates. This limits the versatility of applications in, e.g., analytical science screening^8,13,20^ where PDMS can react with solvents, decreasing the sensitivity of assays and subsequent manipulation or printing due to the stickiness of PDMS.

Khanh and Yoon^15^ applied a polymer layer, polyethylenimine (PEI) with Young’s modulus *Y* ≈ 1 GPa, on physically templated silicon substrates to assemble ordered arrays of silica nanobeads by manual rubbing successfully. However, a post-treatment step was included, which involved calcining at a temperature of 500 °C to remove the layer of PEI, rendering this method inapplicable to assemble polymer particles, e.g., polystyrene, with lower melting temperatures.

In landmark studies,^28−32^ the Whitesides group showed that crystallization of agitated (sub)mm-sized spheres, i.e., the self-assembly of macroscopic bodies by shaking, can be achieved solely through Coulombic electrostatic attractions among the macroscopic spheres. Under ambient conditions, these electrostatic attractions stem from the contact electrification or tribocharging phenomenon, which embodies the process of exchanging charged species when materials are brought in frictional contact and separated.^12,33^ Although this phenomenon also occurs when colloidal particles are rubbed, previous studies^15,16,25,26^ excluded the tribocharging effect on assembling these monolayers comprising closely packed crystals of colloidal particles. However, recently, Jimidar et al.^34^ reported that the tribocharging effect could be leveraged to produce segregation of randomly arranged, i.e., no dominant ordered crystal structures, monolayers comprising silica microspheres on fluorocarbon-coated substrates after rubbing with a PDMS stamp, i.e., particles rubbed between two dissimilar materials.

In contrast to the studies conducted by the Whitesides group using macroscopic bodies, it is more challenging to study the self-assembly of micro- and nanoparticles as the cohesive surface interaction forces (van der Waals, capillary, contact mechanics, and electrostatic contributions) among the particles are significantly high due to their high surface-to-volume ratio.^12,27,35^ Particularly for this reason, scientists have mostly employed the already mentioned wet assembly methods to circumvent these strong cohesive forces.^13,14^ Therefore, given the complexity of all of these different surface interaction force contributions on this scale, a great knowledge gap remains in the physical phenomena involved in dry assembly as in-depth studies are lacking.

In an attempt to close this knowledge gap in the dry assembly of particles and to overcome the challenges in fabricating physically structured substrates or the incompatibility of substrates in applications or assembling polymer particles, we focus here on the rapid (<20 s) manual rubbing assembly of hexagonal closely packed (HCP) crystal structures of monodisperse dry powders on nonelastomeric rigid substrates with a Young’s modulus ranging between 21 and 89 GPa (orders of magnitude larger than PDMS) using a PDMS stamp by accounting for all relevant surface interaction forces. Our radically new rubbing assembly approach is mainly driven by tribocharging and contact mechanics to generate a sufficient amount of adhesion between particles and rigid substrates (*Y* > 20 GPa) as opposed to other rubbing studies. Therefore, by varying the mechanical and electrical properties of the respective substrates, we explore their effect on attaining HCP structures comprising silica, poly(methyl methacrylate) (PMMA), or polystyrene dry colloidal powders with sizes ranging from 500 nm to 10 μm. From our results, we can extract that the powder should comprise free individual particles (“loosely packed”) that can roll during the assembly process and assemble into HCP crystals, and the rubbing stamp should be an elastomeric surface (Young’s modulus on the order of a few MPa) that can tribocharge the system and capture particles on their surface, leaving an assembled monolayer on the rigid substrates intact. Next to those two conditions, we find that the combination of particle and substrate material is key for attaining HCP structures as tribocharging and surface deformations are the main promoting factors to generate sufficient adhesion between the particles and substrates. Therefore, we find that the performance of fluorocarbon-coated substrates surpasses that of the other substrates in assembling HCP crystals. Additionally, the softer polymer particles (*Y* ≈ 3 GPa) assemble in HCP structures on almost every substrate except the silicon and ITO-coated sample, whereas HCP structures of silica particles (*Y* = 74 GPa) were obtained only when the cohesive capillary interactions among them were reduced under zero-humidity glovebox conditions. At last, we show that in a controlled manner, any desirable pattern of HCP crystal structures can be achieved on fluorocarbon-coated SiO_2_ wafers, i.e., chemically patterned wafers, in combination with the application of pressurized air, paving the way for microelectronics,^5^ flexible electronics, electrochemistry,^4^ and (bio)sensing applications^36^ and anticounterfeiting.^37^

## 2 Experimental Section

### 2.1 Materials and Methods

**Figure 1:**
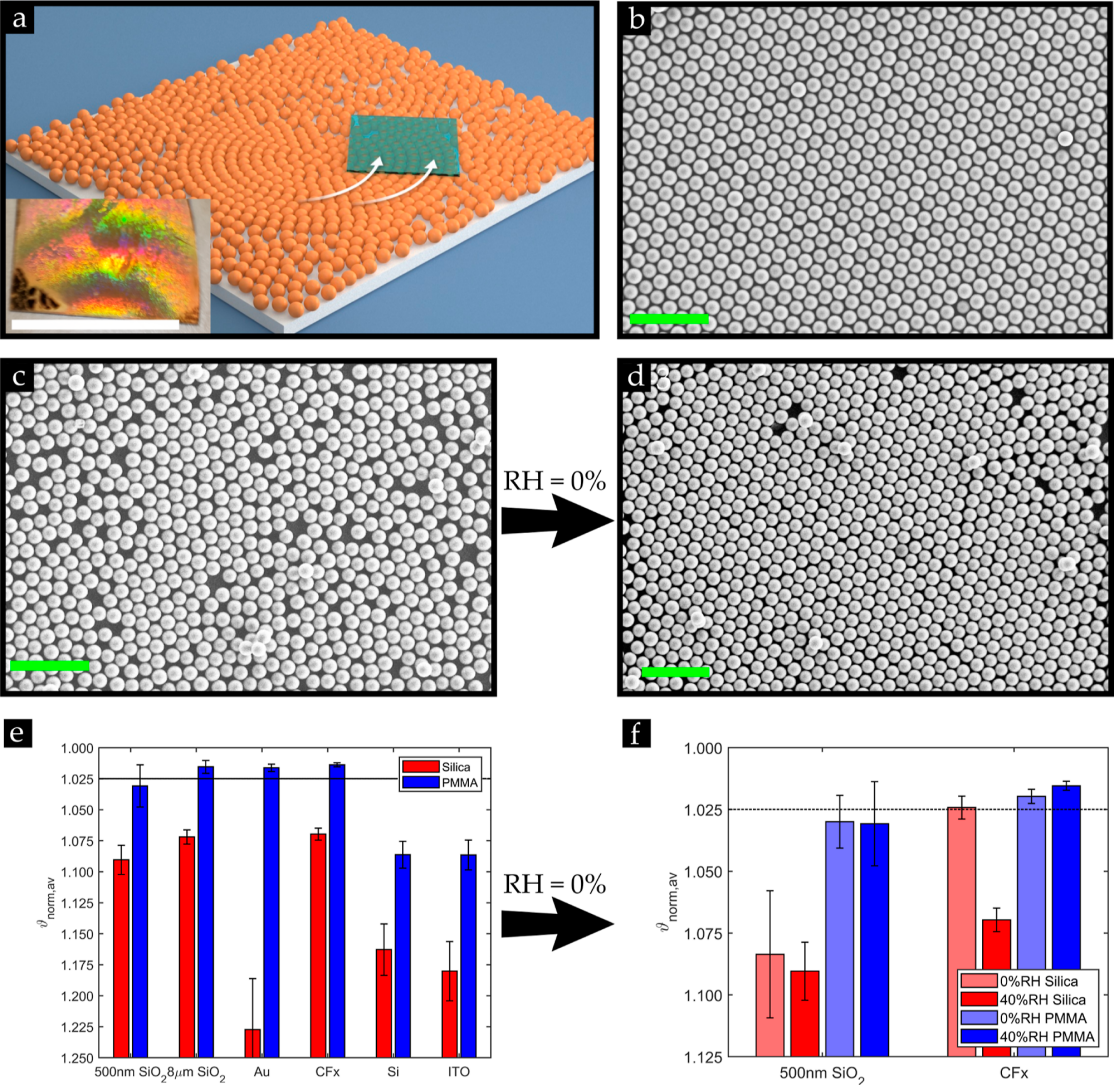
(a) Schematic illustration of the manual rubbing technique to assemble dry powder into closely packed crystal (HCP) structures on substrates using a PDMS (rubber) stamp; inset shows the iridescence structures observed of a 3 μm PMMA monolayer on a fluorocarbon coating on a Au-coated substrate under illumination (scale bar: 2 cm). SEM images of the uniformly fluorocarbon-coated silicon substrate covered with a monolayer of 10 μm (b) PMMA and (c) silica microspheres rubbed under standard lab conditions (RH = 40–55%) and (d) silica microspheres rubbed under zero-humidity glovebox conditions (RH = 0%). (e) Average normalized shape factors ϑ_norm_ of the monolayers obtained on various substrates under standard lab conditions (RH = 40–55%; *N* = 40). (f) Average normalized shape factors ϑ_norm_ of the monolayers obtained under zero-humidity glovebox conditions (N_2_-controlled environment) and standard lab conditions [RH = 40–55% (data taken from Figure 1e); *N* = 40]. Scale bar, green: 50 μm.

The rubbing experiments, illustrated in Figures 1a and S2, were performed using dry powder (15 ± 3 mg in most experiments) of monodisperse spherical silica (diameters of 0.560 ± 0.02 and 10.02 ± 0.32 μm), PMMA (diameters of 0.499 ± 0.010, 3.04 ± 0.11, and 9.95 ± 0.22 μm), and polystyrene particles (diameter of 9.87 ± 0.12 μm) that were all purchased from microParticles GmbH. The manufacturer supplied standard deviations on the particle diameter. Young’s modulus *Y* of the different particle types was provided by the manufacturer: silica (*Y* = 73.6 GPa), PMMA (*Y* = 3 GPa), and polystyrene (*Y* = 3.3 GPa).

It is noteworthy that three persons performed the manual rubbing experiments (which are elaborately described in Section S2) on separate days to minimize the influence of variable rubbing parameters (e.g., applied pressure and rubbing speed) between operators on the quality of the assembled monolayers. The rubbing experiments were performed using rubbing speeds of approximately 2–5 mm s^–1^ (on the small samples of 14 × 14 mm^2^) and 1–3 cm s^–1^ (on experiments performed on wafer-scale). Regarding the manually applied pressure *P* during the rubbing procedure, the operator can distinguish two extreme cases: if the rubbing pressure is too high, particles are removed from the substrate, damaging the assembled layers on the substrates; however, when the pressure is low, particles are not spreading on the substrate, inhibiting the formation of assembled layers. Thus, these indications are key observations for the operator to adjust the rubbing pressure, *P* if necessary, during this manual assembly method.

The rubbing of dry powder was performed on six different substrates: boron-doped p-type silicon (Si) wafers covered with a 2 nm native oxide (Si-Mat), the same p-type Si-wafer with a 500 nm thick oxidized layer (applied by means of wet thermal oxidation at 1150 °C for 36 min), p-type Si-wafers with an 8 μm thick oxidized layer (KST World Corp.), Si-wafer with a 200 nm gold layer (applied by sputtering), and borosilicate glass (MEMpax) wafers with an indium tin oxide (ITO) layer (applied by RF sputtering (=a gas flow of 0.5 sccm O_2_ and 45 sccm Ar was used during the sputtering process, for a total sputtering time of 30 min)), and wafers coated with a fluorocarbon (CF_*x*_) layer. The CF_*x*_ layer (2 ≤ *x* ≤ 3) was deposited on the substrates by plasma polymerization of CHF_3_ in a reactive ion etcher (RIE) system (25 sccm CHF_3_, 11 W, 130 mTorr, 8 min, electrode temperature 20 °C). The fluorocarbon-patterned wafers were manufactured by a standard lithography process, followed by a plasma polymerization process, and the subsequent lift-off of the photoresist from the wafer, as elaborately described by Jimidar et al.^34^

If not stated otherwise, PDMS stamps are used. The PDMS (10:1 w/w) stamps were made by pouring PDMS (SYLGARD 184 silicone elastomer kit; Dow, Inc.) into a Petri dish and cross-linking it in an oven at 65 °C for at least 4 h. The PDMS is eventually cut into pieces of 1 × 1 cm^2^.

A second stamp was introduced consisting of an aluminum film. Aluminum foil (thickness 0.024 mm) was cut to pieces of around 2.5 × 2.5 cm^2^. The individual pieces of aluminum foil were then manually wrapped around a piece of PDMS. It was made sure that at one side of the PDMS piece, the aluminum would make a smooth and tight cover.

A third stamp was composed of a material recently developed by Šutka et al.^38^ The obtained material was composed of polyether block amide (PEBA) and goethite (α-FeOOH); (PEBA)/α-FeOOH is a thin yellow-looking flexible film. A piece of the material was cut, only slightly larger than the PDMS piece. The PEBA/α-FeOOH piece was then placed on a piece of PDMS, and due to its sticky nature, it attached to the PDMS. For obtaining a composite, the goethite α-FeOOH powder was dispersed in chloroform containing 40 mg mL^–1^ PEBA as a stabilizer. After a stable colloidal goethite suspension was obtained by ultrasonication (2 min, 40 W, Hielscher UP200 St ultrasonic processor), the additional PEBA was added to achieve the desired α-FeOOH concentration in PEBA (5 vol %). The solution was stirred and cooled to ambient temperature for 1 h. Afterward, it was poured into a Petri dish and kept at ambient temperature for 3–4 h until the solvent was evaporated. The thickness of the prepared polymer composites was ≈300 μm.

Most of the rubbing experiments were conducted under ambient lab conditions (*T* = 21 ± 1 °C, RH = 40–55%). A set of experiments were conducted under a controlled N_2_ environment inside a glovebox (*T* = 21 ± 1 °C, RH = 0 ± 1%). The temperature and humidity are measured with a Digital Professional Thermo-Hygrometer KLIMA BEE, TFA, Germany. Particles, as well as substrates, were placed in the glovebox environment at least 12 h prior to conducting the rubbing experiments, ensuring the removal of water layers on the surfaces.

### 2.2 Characterization and Visualization

Scanning electron microscopy (SEM) images were taken with a ZEISS Merlin high-resolution scanning electron microscope, while optical microscopy images were collected using a Leica DM2500 MH microscope connected to a ZWO ASI294MC Pro camera.

For each of the particle–substrate combinations investigated under different conditions, i.e., humidity (Figure 1c,d) and rubbing stamp (Figure 3a), experiments were performed on 8 samples of 14 × 14 mm^2^. Subsequently, five images were taken randomly from each of the utilized samples; i.e., each bar represents the data after analyzing 40 images. The quality of the assembled monolayer structures was assessed by employing the Voronoi tessellation approach, which is described in more detail in Section S3.

Kelvin probe force microscopy (KPFM) measurement details are described in Section S7 and can be found in previous reports.^34,39^

Force spectroscopy (FS) was performed with a dimension icon atomic force microscope (Bruker) to obtain force–distance curves (*F*(*D*)). In this mode, the colloidal probe performs an approach and a retraction cycle. It enables precise control over the applied loading force (*F*_L_) and the approach velocity (*v*_a_, which is equal to the retraction velocity). The measurements were performed with PMMA colloids with a diameter of 10 μm (CP-NCH-PM by sQube, NanoAndMore) and with highly boron-doped diamond tips (FM-LC; Adama Innovations Ltd., resistivity: 0.003 to 0.005 Ω cm). From the retraction curve, the different force components are extracted as well as mechanical properties such as Young’s modulus. An elaborated description of the measurement is given in the Supporting Information in sections S5−6. The interested reader is referred to ref (40) for extracting the electrostatic force from *F*(*D*) spectroscopy.

## 3 Relevant Interaction Forces in Dry Assembling Colloidal Particles

As ultrafine dry powder (particles with a diameter <10 μm) is utilized in this study, it should be remarked that these particles exhibit a relatively large surface-to-volume ratio, leading to strong, cohesive interaction forces over body forces, i.e., gravity can be neglected.^35^ In principle, overcoming the surface interaction forces between particles and substrates is the key challenge in dry assembling ultrafine powder into crystal structures.^12,19^ Next, the relevant interaction forces are qualitatively presented. For a more elaborate description, the reader is kindly referred to other reports.^27,35,39^

Broadly taken, the surface interaction forces among two bodies constitute different contributions^27,35,39,41^
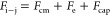
1in which *F*_cm_ is the contact mechanics force, including the van der Waals force and the *F*_e_ electrostatic force, while *F*_cap_ denotes the capillary force. Note that *F*_*i*–*j*_ reflects *F*_p–s_ when particles and substrates are involved, and *F*_p–p_ for the interparticle surface interactions.

The capillary force is particularly a dominant contribution when hydrophilic materials are involved as the present water layers on the bodies form a liquid meniscus between neighboring bodies. This implies that the contribution of the capillary force cannot be neglected when the experiments concern silica powder, silicon, SiO_2_, and ITO-coated substrates.

On the other hand, the van der Waals force, originating from electromagnetic interactions between neutral molecular dipoles, is, in practice, significantly lower than the predicted Hamaker model (considers atomically smooth surfaces) due to the naturally present roughness on the bodies.^27,35^ The materials employed in this study are not atomically smooth but carry a roughness of a few nm and thus can be neglected with respect to all other more dominating contributions given in eq 1.^39^

The contact mechanics force stems from van der Waals interactions and also accounts for elastic deformations at the interface between two contacting bodies, e.g., Hertz, DMT, and JKR theory.^27^ Therefore, Young’s modulus, which is a measure of the elasticity of a solid body, is an important parameter in this regard. Apart from the contact force and the size of the bodies in contact, the established contact area also depends on the effective Young’s modulus of the two bodies.^27^ Consequently, a larger contact area is realized when more elastic or softer materials with a relatively low Young’s modulus get into contact, increasing the contact mechanics force. However, when stiff materials with a higher Young’s modulus are involved, the contact area and adhesion are smaller.^27^

The tribocharging phenomenon, on the other hand, is an interfacial process in which two bodies exchange electrical charges when rubbed past each other,^12,33,42^ leading to the potential onset of an electrostatic force when the particles are rubbed across the substrates. In this regard, the empirically established triboelectric series guides scientists in modern days in predicting the direction of charge transfer between bodies of different materials. The series ranks positively charging materials on the top, while materials gaining a more negative polarity can be found at the tail of the series.^12,43^

## 4 Results and Discussion

Figure 1a depicts a schematic representation of the assembly method in which dry powder is sandwiched between a PDMS stamp and another substrate to attain a monolayer encompassing HCP crystal structures. This is achieved when the powder is manually rubbed in a circular motion across the substrates for approximately 20 s until the sample is entirely covered with particles as shown in Figure S2.

In our initial experiments performed using silica and PMMA microspheres on uniformly fluorocarbon-coated silicon substrates, particle monolayers were formed as shown in Figure 1b,c, which is consistent with our earlier studies in which silica or polystyrene microspheres were involved in manual rubbing^34^ or horizontal shaking^39^ experiments. What immediately stands out from these SEM images is that the PMMA monolayer is conspicuously occupied with HCP crystals, whereas these domains are scarce as far as the silica microspheres are concerned. Starting from this observation, we primarily utilized monodisperse silica and PMMA dry powder microspheres to investigate their distinct behavior in forming HCP structures on pristine, fluorocarbon-coated, or fluorocarbon-patterned substrates, as can be noticed from Figure 1e. Even though HCP structures can be clearly observed with the naked eye from these SEM images, we exploit the Voronoi tessellation approach (cf. Section S3) to quantify the differences between the morphology of the attained monolayers.^39,44,45^ This technique allows us to identify the symmetries existing in particle monolayers, particularly the hexagonal symmetric HCP structures that we seek. Therefore, the shape factor of each individual Voronoi cell is normalized to that of an ideal regular hexagonal cell (ϑ_hex_ = 1.1027), as elaborated in Section S3, i.e., ϑ_norm,hex_ = 1.

A striking observation that can be made from Figure 1e is that for all investigated substrates, ϑ_norm_ is lower for the PMMA powder than for the silica particles, implying that HCP structures predominantly exist on the substrates covered with PMMA microspheres, which coincides with observations made in Figure 1b,c on the fluorocarbon-coated substrate. Thus, these results imply that the particle type plays a key role in the assembly of HCP crystals.

Therefore, we first examine the initial states of the dry silica and PMMA powder. From Figure S1, it is understood that the silica powder comprises massive bonded aggregates, even resembling crystal-like structures, while the PMMA particles comprise only a few smaller aggregates and many free single particles, implying that the cohesive interactions among the hydrophilic silica particles are stronger compared to less hydrophilic PMMA powder.^39^ The large aggregated structures present in the hydrophilic silica powder pose an immediate challenge in the assembly process as they require the application of a sufficiently strong shear force *F*_shear_ that should be transferred through the aggregate during the rubbing motion to mobilize or fluidize the massively aggregated silica powder into free individual particles.^22^ Simultaneously, particles in contact with the substrate should remain on the substrate, which occurs when the particle–substrate interaction force *F*_p–s_ surpasses the particle–particle interaction force *F*_p–p_.^39^ Next, for the available free single spherical particles to form an HCP crystal structure, Park et al. postulated that they should be able to experience a rolling motion while continuously undergoing collisions during the assembly process.^25^ Sliding particles disrupt already assembled crystal structures, mimicking the process of a billiard game.

Thus, the observations in Figure 1b,c,e imply that, in contrast to the PMMA powder, the applied shear force during the rubbing motion was insufficient to overcome the strong capillary interactions among the massively aggregated hydrophilic silica powder, i.e., a stronger shear force *F*_shear_ is required to crush the strongly bonded crystal-like silica aggregates shown in Figure S1a into the required free individual particles,^39^ hindering the formation of rolling single particles into perfect HCP crystal structures during the rubbing motion. A similar observation was previously made, where it was found that the cohesive silica powder required more energy than the polystyrene powder in order to be fluidized.^39^

To eradicate the water content from the silica powder and thus reduce *F*_p–p_ among the silica particles such that the aggregate can be easily crushed into single particles,^40,41^ we repeated the experiments in a zero-humidity glovebox. As a consequence, Figure 1d shows that we can assemble the silica powder into pronounced HCP structures, albeit with some defects, on the fluorocarbon-coated substrates inside of the glovebox. This result emphasizes the necessity of a loosely packed dry powder, i.e., a powder containing free individual particles, that can roll across the substrate to assemble HCP structures eventually. However, despite their more loosely packed state, it appears that improvements have failed on the SiO_2_ substrate within the glovebox (cf. Figure 1d). In addition, from Figure 1d, it can be inferred that regarding the PMMA dry powder, similar results were obtained in the controlled zero-humidity environment as under normal lab conditions. This result was anticipated as the PMMA powder is loosely packed such that the glovebox has a limited effect on this less hydrophilic powder.

Overall, these results elucidate that apart from the necessity of the powder to comprise free individual particles,^22^ and, concurrently, the particle’s ability to roll,^25^ the substrate clearly also matters to what extent HCP crystals will be assembled. The latter signifies the importance of different surface interaction forces at play during assembly.

Regarding the particle’s ability to roll across the substrate during assembly, it is known that spherical particles experiencing a pressure *P* and shear force *F*_shear_ during the rubbing process will perform a pure steady-state rolling motion across the substrate when the rolling friction coefficient μ_r_ satisfies the following condition^25^
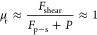
2

Thus, it is implied that particles should experience sufficient friction, which can be tuned by not only controlling the rubbing process parameters, such as the shear force and pressure as reported by Park et al.,^25^ but also the particle–surface interaction forces that play a crucial role in assembling crystal structures using the manual rubbing method. The latter is thoroughly investigated in the remainder of the study.

### 4.1 Various Substrates under Ambient Conditions

The degree to which the contributions in eq 2 will dominate depends on the particle and substrate’s electrical and mechanical properties. To this end, we employed six different substrate samples (14 × 14 mm^2^) to investigate their influence on attaining HCP crystal structures of PMMA and silica powder particles by performing eight experiments for each particle–substrate combination. From each sample, five images were taken to evaluate the quality of the assembled monolayer on the respective substrate using the Voronoi approach (cf. Section S3).

To discriminate the contact mechanics force between the different substrates, we performed nanoindentation measurements using atomic force microscopy (AFM) to determine Young’s modulus of the respective substrates, as thoroughly described in Section S6. The values of the analyzed Young’s modulus of each substrate are presented in Table S1. We categorize the substrates with a relatively high Young’s modulus as stiff, while other substrates are more elastic materials. The former type of substrate can be less deformed upon contact, generating a lower contact mechanics force compared to the elastic materials.^27^

Another immediate observation that can be made from the data presented in Figure 1d,e is that, on average, none of the combinations results in ϑ_norm_ = 1, which implies that a perfect single crystal is absent from the substrate, i.e., the monolayers are not monocrystalline and thus contain defects, such as grain boundaries, vacancies, or a few excess particles, which is common for two-dimensional systems.^2^ Additionally, it can be observed from Figure S5 that the quality of the monolayers is similar for areas in the center and edge of the substrates with a few more grain boundaries existing when moving away from the center. These findings can be explained by considering the circular rubbing motion during the assembly process, indicating that the PDMS rubbing stamp frequently moved in the sample’s center, which recovers defects.^25^

In Section S3, examples are provided in Figures S3 and S4 for different average values of ϑ_norm_ to guide the reader when interpreting the data reported for the average obtained shape factors. From Figures S3 and S4, we define the condition that 1 ≤ ϑ_norm_ ≤ 1.025 to consider that an HCP-ordered monolayer was attained.

#### 4.1.1 Nonconducting Substrates

Figure 1e shows that despite the large aggregates present in silica powder (cf. Figure S1a), the best results are obtained on the fluorocarbon-coated substrate followed by the SiO_2_ substrates. From our preceding work using KPFM^34,39^ and colloidal probe measurements,^40^ and the triboelectric series,^12,33^ we already know that the silica microspheres are prone to exchange electrical charge with the fluorocarbon-coated surfaces by means of the triboelectric charging phenomenon, such that the particles and fluorocarbon layer acquire opposite charges, inducing an attractive electrostatic force *F*_e_ between them. This ensures that a monolayer of silica microspheres is firmly captured, however nonclosely packed (cf. Figure 1c). This is plausibly due to strong particle–particle cohesive interactions among the microspheres, which causes a frustrated moving state that inhibits a rolling motion needed to form dense, closely packed crystal structures.^39^

As the SiO_2_ substrates also pertain to the electrically insulating substrates, it is anticipated that they also can get charged during the rubbing process, but as the silica microspheres and SiO_2_ substrates are of a similar chemical nature, it is expected from the triboelectric series that charge transfer will be limited. However, KPFM measurements verified that the contact potential difference of the SiO_2_ substrates was increased after they were rubbed with silica microspheres using PDMS stamps. It is plausible that the SiO_2_ substrates are charged as a result of the material transfer from the PDMS slab during the rubbing process. It is known that due to the heterolytic bond cleavage, the PDMS surface transfers charge,^46^ which is constantly occurring during the rubbing motion of particles on the substrate.

Despite the existing electrostatic attraction, the results shown in Figure 1e attained using the silica microspheres on the SiO_2_ substrates are inferior to (or of lesser quality than) those on the fluorocarbon-coated substrates. Additionally, the average normalized shape factor presented in Figure 1e shows that the formation of crystal structures is promoted as the thickness of the oxide layer increases. The latter can be attributed to an approximately two times lower Young’s modulus *Y* (cf. Table S3) of the 8 μm thick oxide layer (*Y* = 34 ± 5 GPa) than the 500 nm SiO_2_ substrate (*Y* = 62 ± 5 GPa). Consequently, a larger contact area is formed between the silica microspheres and the 8 μm SiO_2_ substrate, concurrently resulting in a stronger adhesion. What is even more than the low Young’s modulus of the fluorocarbon-coated substrate (*Y* = 21 ± 5 GPa) is the strong tribocharging-induced electrostatic attraction that generates sufficient adhesion for the silica microspheres to remain on the substrate, favoring the formation of crystal structures on fluorocarbon-coated surfaces. The latter is in agreement with the observations made from the experiments inside the zero-humidity environment (cf. Figure 1d,f), where HCP crystals of the silica microspheres were present on the fluorocarbon-coated substrate but not too often on the SiO_2_ substrate. The fact that the tribocharging-induced electrostatic attraction on the fluorocarbon-coated substrate is stronger in comparison to the SiO_2_ substrates is corroborated by our recent colloidal probe findings.^40^

Of course, intuitively, one expects that for both of these hydrophilic SiO_2_ substrates and silica powder, the capillary force may also dominate the adhesion force under ambient conditions. Therefore, we also performed experiments on a hydrophilic silicon substrate carrying a 2 nm native oxide layer, which is stiffer than that of the 500 nm SiO_2_ substrate. Previously, we already reported that no change in the contact surface potential could be measured on pristine silicon substrates after rubbing or shaking silica particles on them,^34,39^ thereby excluding strong enduring electrostatic attractions. The data in Figure 1e indicate that the capillary force between the silica particles and substrates has a negligible effect as the pristine silicon substrate is the most unfavorable from the surfaces discussed above to attain HCP structures of silica microspheres.

As noted before, clear, distinctive results were obtained using PMMA microspheres in contrast to the silica powder. Figure 1 shows that the PMMA microspheres predominantly formed HCP structures on the uniformly fluorocarbon-coated surface (cf. Figure 1e) as well as on the pristine SiO_2_ substrates. The triboelectric-induced attraction and the contact mechanics force are plausibly the main constituents of the adhesion force, *F*_p–s_, for these less hydrophilic powders.

**Figure 2:**
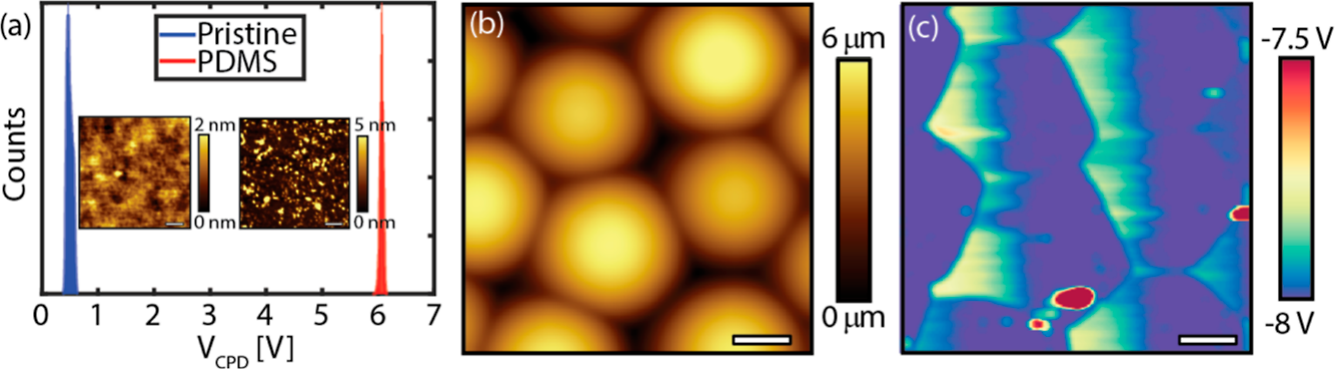
Results of the KPFM measurement performed on the (a) 500 nm SiO_2_ substrate before (pristine) and after rubbing the PMMA microspheres using a PDMS stamp. Inset (a) shows the topographic image (7 × 7 μm^2^, scale bar: 1 μm) of the SiO_2_ substrate. (b) Topographic (25 × 25 μm^2^, scale bar: 6 μm) and (c) simultaneously obtained surface potential map (25 × 25 μm^2^, scale bar: 6 μm) of the 10 μm PMMA microspheres after the rubbing experiment.

The triboelectric charging is supported by KPFM measurements performed on the PMMA microspheres and their respective substrates. Note that an increase in contact potential difference *V*_CPD_ corresponds to a more acquired negative charge, whereas a positive polarity matches a negative *V*_CPD_ value. Without a doubt, Figure 2 shows that after rubbing, the pristine 500 nm SiO_2_ substrate acquires a more negative charge, implying that the substrate captures negatively charged species during the rubbing procedure, while the PMMA microspheres acquire a positive charge. The same holds for KPFM measurements performed on the other two substrates. Thus, an electrostatic attraction is induced between the positively charged particles and the negatively charged substrates, adding to the adhesion force *F*_p–s_. It can also be inferred from Figure 2c that the PMMA particles have the same polarity, which would imply that a Coulombic repulsion (cf. Section 3) between the microspheres would counter crystal formation. Thus, this result signifies that the electrostatic attraction between the PMMA microspheres and the underlying substrate is sufficiently strong to overcome the repulsive force, enabling the assembly of the HCP crystals.

From Figure 1e, it can be remarked that the error bar of the average shape factor of the assembled HCP crystals on the 500 nm SiO_2_ substrate has a larger error bar with respect to the other two substrates. It is safe to assume that as the 500 nm SiO_2_ substrate is stiffer (*Y* = 62 ± 5 GPa), smaller deformations occur at the interface, and concomitantly, the PMMA particles experience lower adhesion compared to the 8 μm Si_2_ (*Y* = 34 ± 5 GPa) and fluorocarbon-coated substrate (*Y* = 21 ± 5 GPa). Note that on the fluorocarbon-coated substrate, tribocharging is also the strongest, which adds to the adhesion.^40^

On the other hand, the monolayers on the pristine silicon substrates contain less pronounced HCP crystal structures of PMMA microspheres, albeit significantly more than the silica microspheres. This result can be ascribed to the lower Young’s modulus of the PMMA microspheres than the silica ones, which promotes the adhesion between PMMA particles and the pristine silicon substrates. Another plausible explanation is that triboelectrification occurs between the PMMA particles and the silicon substrate. However, as the KPFM measurements are measured about 10 min after the rubbing experiment, all electrostatic charges may have dissipated from the silicon substrate, and therefore, no change in the contact potential difference can be detected. This is supported by our colloidal probe study in which contact electrification between a polymer sphere and a silicon substrate was measured within a second.^40^ In addition, the charging of PMMA particles due to the PDMS stamp, i.e., charging between two polymers by means of material transfer,^46,47^ cannot be excluded.

#### 4.1.2 Conductive Substrates

To minimize electrostatic attraction between particles and underlying substrates, we performed experiments on conductive substrates: a silicon surface uniformly covered with a 200 nm Au layer and an ITO-glass substrate, as displayed in Figure 1e. Using KPFM, we verified that the contact surface potentials of these respective substrates also remained unchanged after the rubbing experiment. Note that as these measurements are performed 10 min after rubbing the particles, it is possible that some charged species are transferred during the rolling motion of particles on the substrates.

Even though the Au-coated substrate’s Young’s modulus (*Y* = 45 ± 5 GPa) is of a similar magnitude as the 8 μm SiO_2_ substrate (*Y* = 34 ± 5 GPa), it is remarkable that the silica microspheres are swept from the Au-coated substrate, let alone form monolayers, whereas HCP structures comprising PMMA microspheres are conspicuously present on the Au-coated substrate as inferred from the average normalized shape factor in Figure 1e. The latter is attributed to the lower Young’s modulus of both Au-coated substrate and PMMA microspheres (*Y* = 3 ± 5 GPa), while the more rigid silica particles (*Y* = 73.6 ± 5 GPa) are less deformable and experience no electrostatic attraction to generate sufficient adhesion to stick on the substrate. However, in the former case, tribocharging of the PMMA spheres by the PDMS stamp also adds to the formation of crystals.^46,47^

On the other hand, it appears that although the ITO-coated substrates (*Y* = 54 ± 5 GPa) are slightly stiffer than the 500 nm SiO_2_ substrate (*Y* = 62 ± 5 GPa), HCP structures are absent from the ITO-coated substrates. This elucidates the necessity of a strong tribocharging-induced electrostatic attraction to generate sufficient adhesion for the powders to stick and form HCP crystal structures on relatively stiff substrates such as the 500 nm SiO_2_ insulating substrate. Regarding the hydrophilic silica powder, the capillary force plausibly allows for some microspheres to stick to the hydrophilic silicon and the ITO-coated substrates under ambient conditions (Figure 1e). It is noteworthy that performing experiments using silica particles on the ITO-coated (*Y* = 54 ± 5 GPa) and silicon substrate (*Y* = 89 ± 5 GPa) under zero-humidity conditions did not improve the monolayer assembly at all due to a lack of tribocharging-induced electrostatic attraction and the stiffness, such that the adhesion between the particles and substrates *F*_p–s_ is too low.

Altogether, these results underscore that having the capacity to generate sufficient adhesion between the particles and substrate (*F*_p–s_) and a rolling motion across the substrate will allow individual free spherical particles to assemble in HCP crystal structures. Evidently, tribocharging-induced electrostatic attraction and the contact mechanics force are the main adhesion contributors, and the capillary force is to a much lesser extent. The data elucidates that the tribocharging phenomenon is crucial in the assembly of HCP structures when either of the actors, particle or substrate, is relatively stiff with a high Young’s modulus (*Y* > 50 GPa) as these stiffer materials fail to establish a sufficiently strong contact mechanics force. Furthermore, the data in Figure 1e,f, particularly those obtained using the silica particles, shows that the strongest tribocharging-induced electrostatic attraction emerges on the fluorocarbon-coated substrate.^34,40^

### 4.2 Different Stamps

So far, all rubbing assembly studies have restrictively utilized either flexible rubbery stamps such as PDMS^16,25^ or bare fingertips.^15,22^ In contrast to earlier work reported by the Jeong group,^16,25^ we explore the assembly of powder sandwiched between two dissimilar substrates, which adds to the complexity of the current contribution. As briefly mentioned, the PDMS stamp can tribocharge the system,^46,47^ promoting crystal formation, as corroborated by our data presented in Figures 1e and 2. To explore the stamp’s effect on the crystal assembly, we employed PDMS wrapped with an aluminum foil (*Y* = 20 GPa) and a polyether block amide (PEBA)/α-FeOOH (*Y* = 30 MPa)^38^ layer covering the PDMS stamp apart from the naked PDMS (*Y* = 1 MPa) applied before (the values are determined using *F*(*D*) spectroscopy and are elaborately discussed in Section S6 in the Supporting Information). The aluminum foil is a conducting surface and, therefore, is not expected to induce much charge. The other two polymer stamps, however, are known to induce charge, which aids the assembly of particles during the rubbing process, as already discussed.

**Figure 3:**
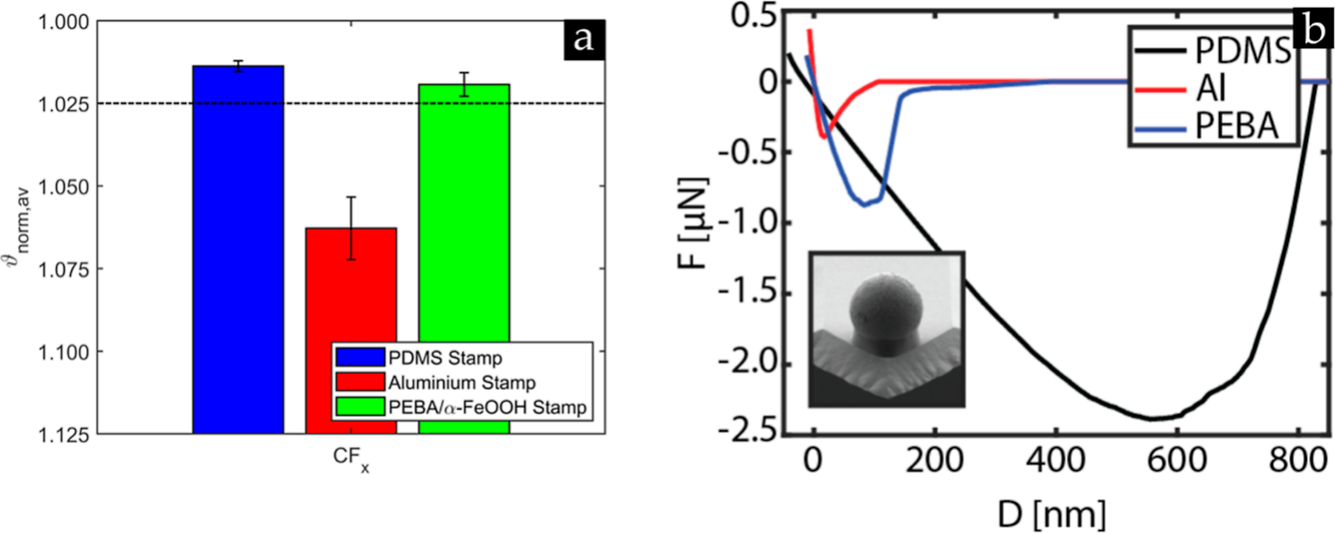
(a) Average normalized shape factor ϑ_norm,av_ of the 10 μm PMMA microsphere results obtained on the fluorocarbon-coated samples (*N* = 40) using three different stamps: pristine PDMS, PDMS wrapped in aluminum foil, and PDMS covered with a (PEBA)/α-FeOOH layer performed under ambient lab conditions (RH = 40–55%). (b) Force–distance curves of a silica colloidal probe on three different stamps obtained using the colloidal probe technique performed with the AFM. Inset (b) displays a silica colloidal probe with a diameter of 10 μm.

The data presented in Figure 3a shows that the performance of the aluminum foil is inferior to the pristine and (PEBA)/α-FeOOH-covered PDMS stamp on all of these investigated substrates. Contrary to the other two types of stamps, the aluminum foil was not covered with powder after the rubbing process, implying that the aluminum foil is less adhesive and deformable to capture a layer of particles. The colloidal probe measurements shown in Figure 3b corroborate this observation, from which it can be readily observed that the colloidal probe experienced limited adhesion and jumped immediately out of contact with the aluminum foil stamp compared to the other two stamps. Consequently, any formation of HCP crystal domains was inhibited at the expense of the aluminum foil-wrapped PDMS stamp, which kept sweeping particles from the samples. This undesirable event could not even be recovered by supplying more powder to increase the probability of collisions among the particles and, concomitantly, the formation of HCP crystals on the samples. The observation of the aluminum foil-wrapped PDMS supports the notion that the bond cleavage of polymers (PDMS and (PEBA)/α-FeOOH) induces triboelectric charging of the system,^46,47^ favoring crystal formation.

Additionally, the results highlight the stickiness of the naked PDMS stamp, as due to its soft elastomeric nature, the colloidal probe remained much longer in contact when retracted from the PDMS stamp. In this respect, it should be mentioned that in all of the investigated cases discussed in Figure 1c, monolayers comprising HCP crystal structures of both silica and PMMA microspheres were attained on the naked PDMS stamps (cf. Section S8 and Figure S8), signifying the strong adhesion established between the particles. This is in agreement with the earlier work from Park et al.,^25^ as due to the stickiness and softness of the PDMS, even silica particles can be captured from the large aggregated structures, such that the PDMS stamp encapsulates the particles, but just enough so that they can still roll and form HCP crystal structures. A similar observation was made on the PEBA-covered PDMS stamps (Figure S8), highlighting that the adhesion is enough to form HCP crystals on the PEBA layer. Also, on the fluorocarbon-coated samples, HCP crystals of PMMA powder were formed using the PDMS stamp covered with the PEBA layer, as shown in Figure 3a.

Thus, from these results, it is inferred that the stamps should encompass the property of tribocharging the system and simultaneously be sticky to capture a monolayer of particles, preventing damaging the assembled HCP crystals on the nonelastomeric substrates.

### 4.3 Other Particle Types and Sizes

**Figure 4:**
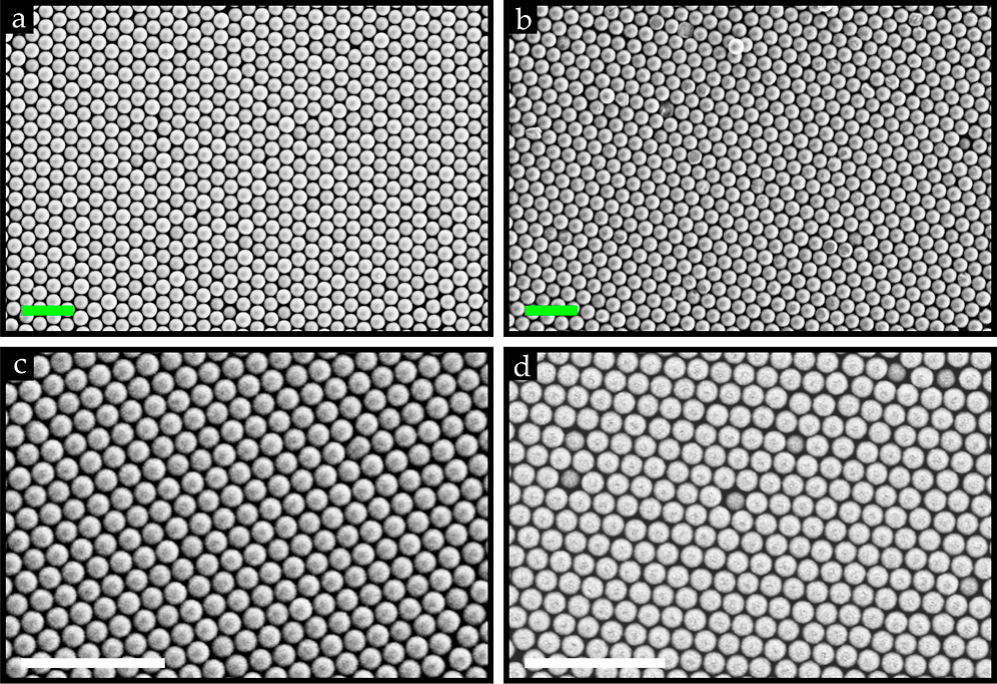
SEM images of HCP crystal structures obtained from 3 μm (a) PMMA powder and (b) polystyrene microspheres and 500 nm (c) PMMA powder and (d) silica powder (inside the glovebox) obtained after rubbing experiments were performed using a PDMS stamp on mainly fluorocarbon-coated substrates. Only experiment (b) was performed on a Au-coated substrate. Scale bar: green = 15 μm; white = 3 μm.

To highlight the versatility of the currently proposed rubbing method on nonelastomeric substrates, we have also explored the assembly of other sizes of PMMA powder particles while also assembling HCP structures from polystyrene microspheres. Figure 4a,c shows that we could successfully assemble PMMA microspheres down to 500 nm on the fluorocarbon-coated substrates. Furthermore, as the initial state of the hydrophobic polystyrene powder is also “loosely packed” (comprises free single particles)^39^ as the PMMA particles, they could also be assembled, for example, on Au-coated substrates as shown in Figure 4b. Similar to the 10 μm silica powder, the 500 nm silica particles were also assembled in HCP crystals on a fluorocarbon-coated surface only if the experiments were performed under zero-humidity conditions. This was expected as the cohesive interactions become relatively stronger when the size of the particles decreases.

Similar to previous rubbing studies on soft substrates, such as PDMS (*Y* ≈ 2 MPa)^25^ or PEI (*Y* ≈ 3 GPa),^15,23^ it remains to be explored in the future if particles with a diameter down to 100 nm can be assembled using the tribocharging-driven rubbing approach presented here on stiffer substrates (*Y* > 21 GPa). However, the challenge will be to overcome the stronger cohesive interactions *F*_p–p_ among these smaller particles and establish sufficient adhesion *F*_p–s_ with the substrates. The latter is less challenging to achieve on the already explored softer substrates by other groups.^15,25^

### 4.4 Tunable HCP Crystal Patterns on Wafer-Scale

Stemming from our earlier findings in which the strongest tribocharging-induced electrostatic attraction was accomplished on the fluorocarbon layer, a new opportunity for assembling tunable patterns of HCP crystals on a wafer-scale emerged.

**Figure 5:**
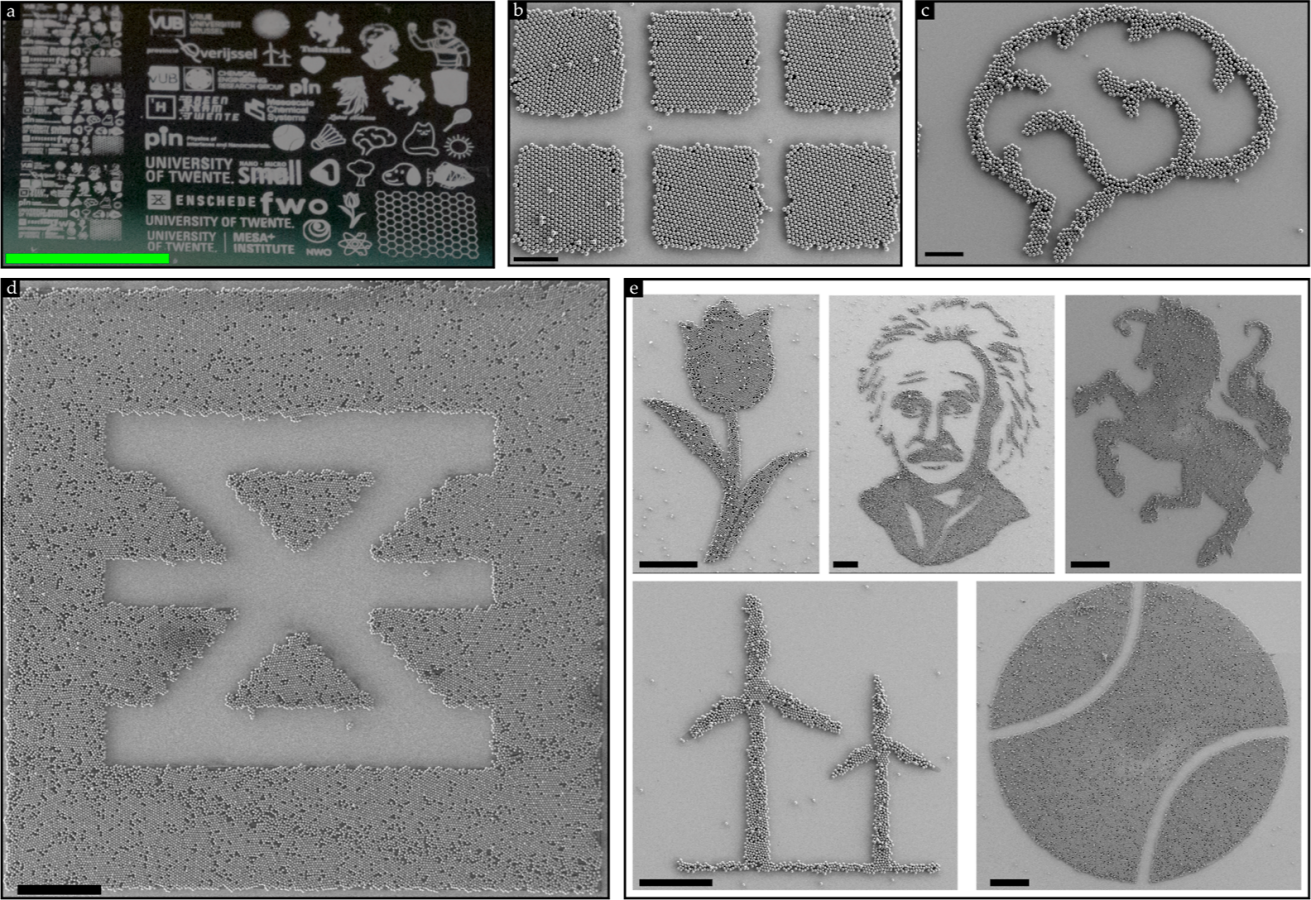
(a) Large area of tunable 2D crystal structures on a 4 in. fluorocarbon-coated patterned 8 μm SiO_2_ wafer after 3 μm PMMA powder was rubbed and subsequently pressurized air of 4–5 bar was laterally blown across the wafer (cf. Figures S9–S11). SEM images of patterned HCP crystal structures obtained using (b,c) 10 μm and (d,e) 3 μm PMMA powder microspheres on the same 8 μm SiO_2_ patterned wafer. The experiments were performed under ambient lab conditions (RH = 40–55%). Scale bar: green = 7 mm; black = 100 μm.

PMMA microspheres of 3 and 10 μm were rubbed on an 8 μm SiO_2_ wafer that was patterned with isolated fluorocarbon patches with a thickness of 50–75 nm. The 8 μm SiO_2_ wafer was chosen to ensure that a monolayer of HCP crystals was formed across the entire wafer, i.e., on the uncoated and coated areas. It could be noticed that the PMMA wafer covered the entire wafer with a monolayer of particles, as suspected from the elaborate preceding discussions. However, by subsequently blowing pressurized air laterally across the wafer, we readily observed that the PMMA microspheres were removed from the uncoated areas of the full SiO_2_ wafer (cf. Figures S9–S11), as shown in Figure 5a. From the SEM images displayed in Figure 5, we can infer that HCP crystals can be obtained on any tunable isolated geometry of a fluorocarbon patch. However, inevitably, pressurized air (4–5 bar) also removed a few particles from the fluorocarbon patterns. Nevertheless, these results underscore the robustness of the tribocharging-induced electrostatic attraction between the particles and the fluorocarbon layer as it is scalable and capable of withstanding the pressurized airflow. Figure S10, including the inset in Figure 1a, shows the diffraction pattern produced by the 3 μm PMMA microspheres, indicating the quality of the assembled colloidal crystals on the macroscopic scale.

The fact that the fluorocarbon layer is able to capture the particles firmly even when blowing pressurized air underscores our earlier findings^40^ that the tribocharging and, concurrently, the adhesion between particles and fluorocarbon coating is the strongest compared to the SiO_2_ substrate. It should be mentioned that a quantitative measure of the triboelectric-induced electrostatic is not possible at this point as a knowledge gap remains in the literature to translate the KPFM data from the particles to actual electrical charge present on the surface.^12^

The findings that the HCP crystal patterns can be tuned on these fluorocarbon-patterned substrates can be advantageous to flexible electronics,^5^ anticounterfeiting,^37^ and solid phases in biochemical reactions or chemical assays.^36^

## 5 Conclusions

To sum up, our results uncover that in designing systems which exhibit strong triboelectrification, dry powders can be rapidly assembled (<20 s) in highly ordered, close-packed monolayers of colloidal particles on various substrates, including nonelastomeric ones (21 < *Y* < 89 GPa) using the rubbing method. In general, triboelectric charging is desirable on both substrates for providing adhesion, particularly when either the particle or substrate is stiff, and rubbing stamp for introducing more triboelectric charging in the system. Based on the latter, naked PDMS and (PEBA)/α-FeOOH-covered PDMS stamps promoted the formation of HCP crystals on rigid substrates. The tribocharging of the system is supported by KPFM measurements, showing that on the fluorocarbon-coated substrates and the SiO_2_ substrate, an electrostatic attraction exists as particles and substrate get opposite polarity after the rubbing process.

The findings elucidate that apart from the tribocharging-induced electrostatic attraction, the contact mechanics force is an essential contributor in generating sufficient adhesion between the particles and various substrates. Therefore, when the substrates are more conductive and less chargeable, they should be more elastic to promote the formation of monolayers comprising HCP crystals. As a final constraint, we find that HCP crystal structures can be attained on rigid substrates solely when experiments are performed with a “loosely packed”, i.e., noncohesive powder containing mostly free individual particles. This aids a pure rolling motion of the particles needed to assemble the aspired structures.

Moreover, the proposed rubbing method using a PDMS stamp is extremely versatile as we were able to assemble HCP crystals of monodisperse silica, polystyrene, and PMMA particles ranging from 500 nm to 10 μm on tribocharged rigid substrates with a Young’s modulus between 21 and 62 GPa, paving the way for analytical screening and particle transfer purposes.

Furthermore, we demonstrated the scalability of the process by assembling arbitrary patterns of crystals on a 4 in. fluorocarbon-patterned, i.e., a chemically templated, wafer, underscoring that the strongest triboelectrification and concomitant adhesion are achieved on the fluorocarbon layer. This efficient, rapid (<20 s), universal, and scalable dry rubbing assembly patterning technique holds promising opportunities for electronic/sensing applications, anticounterfeiting, solid supports for biochemical reaction applications, and other aspects.

## Supporting Information Available

Additional experimental details, Voronoi approach to quantify the quality of the assembled monolayers, force spectroscopy measurements, determining Young’s modulus, Kelvin probe force microscopy details, monolayers assembled on the different stamps, and wafer-scale assembly of HCP crystals on tunable patterns and iridescence structure colors observed under illumination (PDF)
