# Supplementary material for: Toward the Assembly of 2D Tunable Crystal Patterns of Spherical Colloids on a Wafer-Scale
Source: ACS Appl Mater Interfaces. 2024 Jan 25;16(9):12007–17. doi: 10.1021/acsami.3c16830 (PMC10921376; doi:10.1021/acsami.3c16830)
Supplement: Supplementary file 1 — am3c16830_si_001.pdf [file am3c16830_si_001.pdf]

Supporting Information

Toward the Assembly of 2D Tunable Crystal Patterns of Spherical Colloids on a Wafer-Scale

Kai Sotthewes,[†] Gijs Roozendaal,[†,‡] Andris Šutka,[¶] and Ignaas S. M. Jimidar*[,§,‡]

†*Physics of Interfaces and Nanomaterials, MESA+ Institute, University of Twente, P.O. Box 217, 7500AE Enschede, The Netherlands*

‡*Mesoscale Chemical Systems, MESA+ Institute, University of Twente, P.O. Box 217, 7500AE Enschede, The Netherlands*

¶*Institute of Materials and Surface Engineering, Faculty of Materials Science and Applied Chemistry, Riga Technical University, LV-1048, Riga, Latvia*

§*Department of Chemical Engineering CHIS, Vrije Universiteit Brussel, Brussels, 1050, Belgium*

E-mail: i.s.m.jimidar@utwente.nl

Contents

1 **Powder particles** **S3**

2 **Rubbing procedure** **S3**

3 **Voronoi analysis** **S4**

4 **Monolayer quality across sample** **S6**

5 $F(D)$**-spectroscopy** **S7**

6 **Determining the Young's modulus** **S8**

7 **Kelvin Probe Force Microscopy measurements** **S11**

8 **Monolayers on different stamps** **S12**

9 **Wafer-scale assembly of tunable HCP crystal patterns** **S12**

References **S16**

1 Powder particles

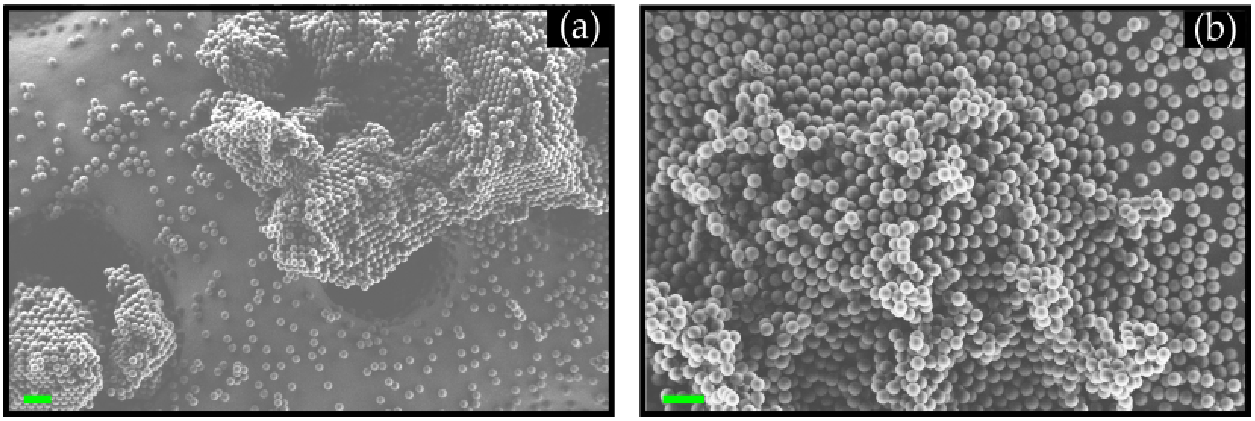

Figure S1: SEM images of the initial state of the as-received 10 μm (a) silica and (b) PMMA powder particles. Scale bar: 30 μm.

2 Rubbing procedure

The particle powder is gently deposited on the middle of the surface of the substrate with a small spoon (Figure S2c). Afterwards, the to-be-used stamp is placed on top of the particle powder (Figure S2d). With the use of a finger on top of the stamp, a homogeneous pressure is applied on the particles, pressing them down on the substrate and breaking the particle agglomerate (Figure S2e). With a circular movement, the particles are then carefully rubbed over the substrate for approximately 20 seconds, until a particle monolayer is created (Figure S2f). It is insurmountable that excess particles spill over the substrate during the process. On a few occasions, an additional amount of particles was added to the substrate after rubbing for a few strokes, for example in cases of very low particle-substrate adhesion.

Because of concerns about the consistency of the rubbing experiments each experiment (with each a different stamp-particles-substrate and environment conditions combination) was performed eight times. These were divided over two consecutive days (four experiments each day) and performed by three different persons to account for slight differences in the surrounding environment and manual rubbing consistency.

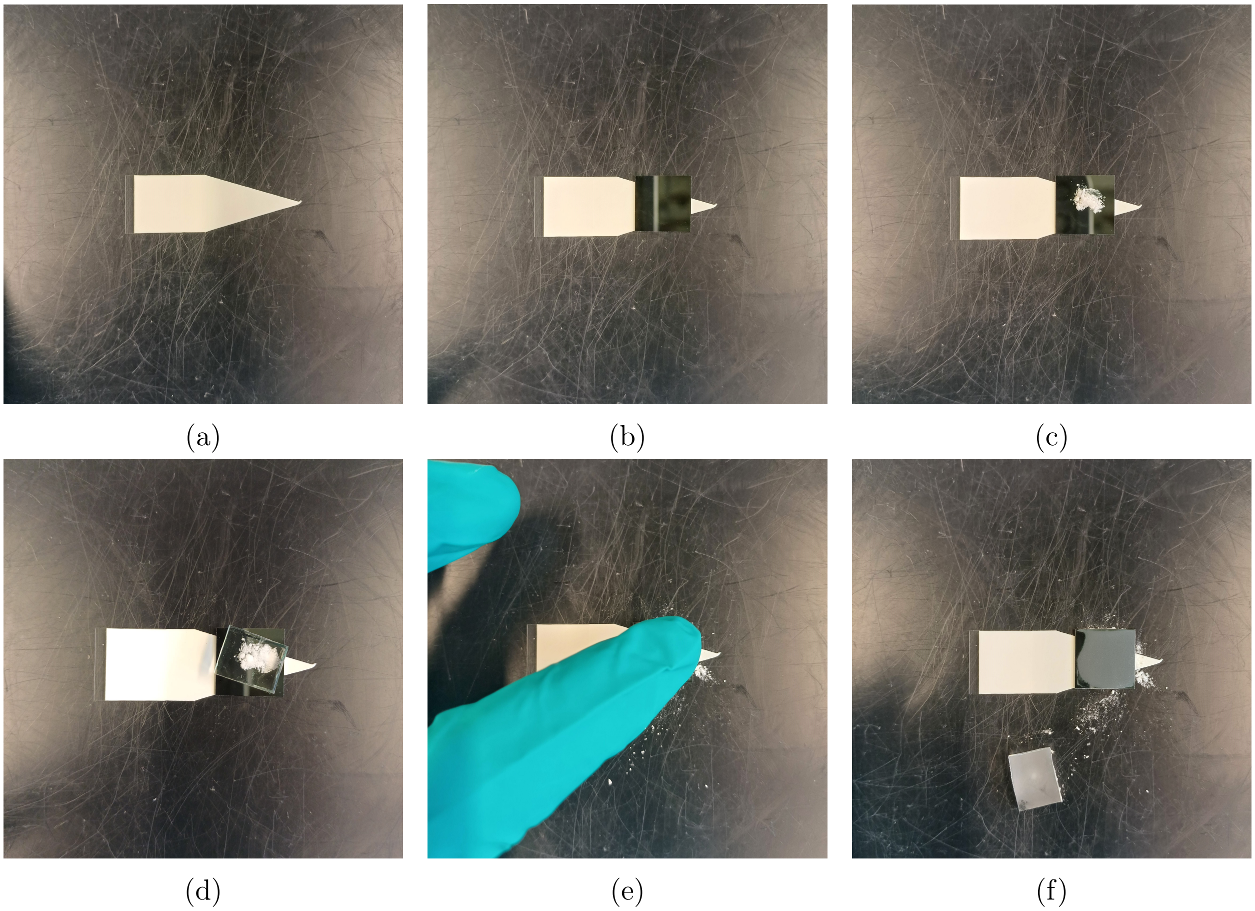

Figure S2: Snapshots of the rubbing method procedure. (a) shows the removable tape on which in (b) the substrate is placed. (c) shows the particle powder deposited on the substrate, after which in (d) a slab of PDMS (the stamp) is placed on top. (e) shows a snapshot of the manual rubbing. Finally, a particle monolayer is formed on the substrate, which is shown in (f).

3 Voronoi analysis

To characterize the structure of the silica or polystyrene monolayers, Voronoi diagrams have been constructed from the optical microscope images using MATLAB routines. A Voronoi diagram, or so-called tessellation, is constructed by using the centers of the microspheres to partition the space into polygonal cells comprising points closer to one microsphere center than to all the others.[1,2] The shape of each obtained Voronoi cell i can be described by

computing a dimensionless quantity ϑ_i, called shape factor:

$$\vartheta_i = \frac{{p_i}^2}{4\pi A_i} \tag{S1}$$

where p_i and A_i are the perimeter and area of Voronoi cell i, respectively. From the definition, it can be concluded that $\vartheta_i = 1$ corresponds to a circular cell, whereas $\vartheta_i > 1$ for regular polygons. In case of a perfect hexagonal crystal, the Voronoi diagrams would result in regular hexagons with $\vartheta_{i,\text{hex}} = 1.1027$.

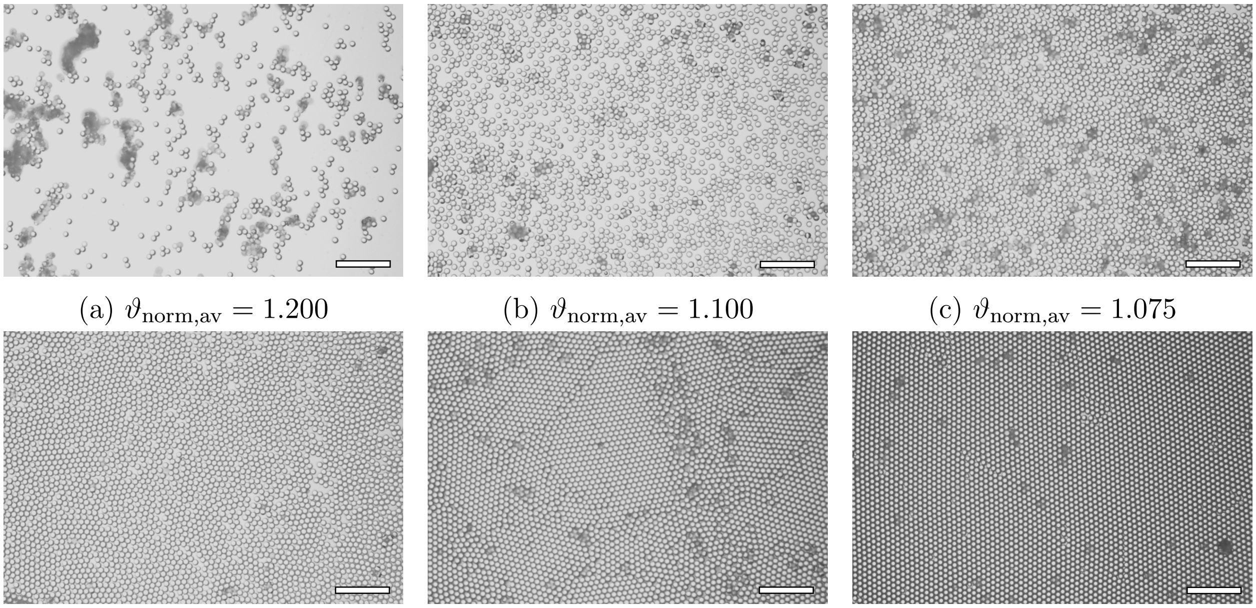

(a) $\vartheta_{\text{norm,av}} = 1.200$ (b) $\vartheta_{\text{norm,av}} = 1.100$ (c) $\vartheta_{\text{norm,av}} = 1.075$

(d) $\vartheta_{\text{norm,av}} = 1.050$ (e) $\vartheta_{\text{norm,av}} = 1.025$ (f) $\vartheta_{\text{norm,av}} = 1.010$

Figure S3: A range of microscope images showing different PDMS rubbing-induced particle assemblies corresponding to different values of the average normalized shape factor ($\vartheta_{\text{norm,av}}$). The scalebar is 100µm. The particle assemblies shown are (a) 10 µm silica on Au, (b) 10 µm PS on Si, (c) 10 µm silica on CF_x, (d) 10 µm silica on CF_x, (e) 10 µm PS on CF_x, (f) 10 µm PMMA on Au.

In figure S3, six particle assemblies are shown after rubbing different particles on various substrates using the PDMS stamp. In addition, the corresponding average normalized shape factor ($\vartheta_{\text{norm,av}}$) is given to give a qualitative number for the quality of order. The figure illustrates that the particle assemblies made with different particle-substrate combinations show different degrees of order, resulting in a different average normalized shape factor, as

calculated by equation S1.

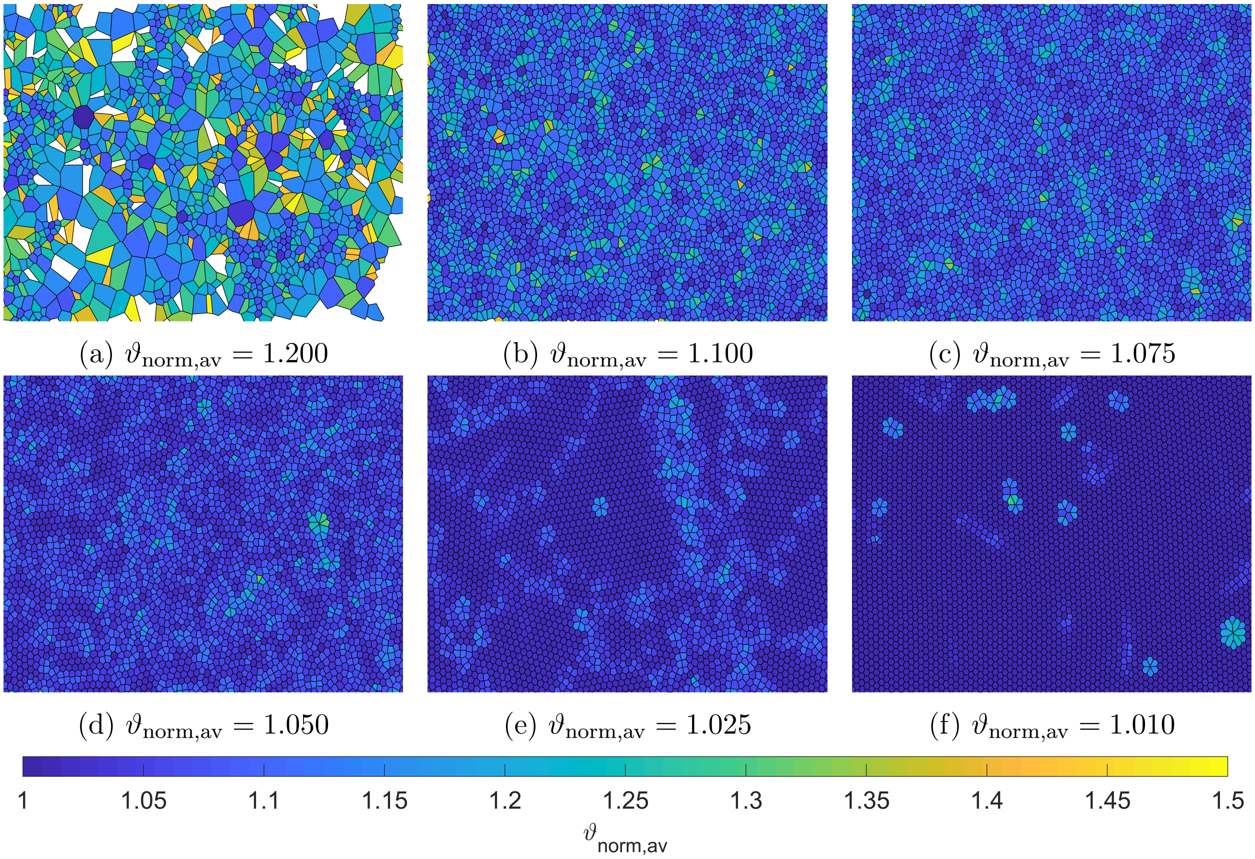

Figure S4: The corresponding Voronoi images of the microscope images displayed in Fig. S2 showing different PDMS rubbing-induced particle assemblies corresponding to different values of the average normalized shape factor ($\vartheta_{\text{norm,av}}$). The particle assemblies shown are (a) 10 µm silica on Au, (b) 10 µm PS on Si, (c) 10 µm silica on CF_x, (d) 10 µm silica on CF_x, (e) 10 µm PS on CF_x, (f) 10 µm PMMA on Au.

4 Monolayer quality across sample

Figure S5 shows the assembled monolayers comprising of 10 µm on the Au-coated substrates. Images were taken from the center to the edge of the sample, and the quality of the monolayers was quantified using the Voronoi approach.

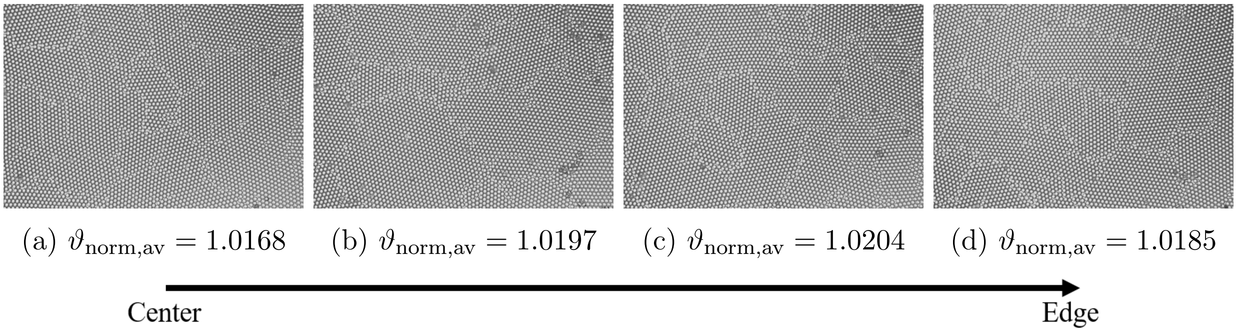

Figure S5: (a-d) Optical microscope images taken from (a) center to (d) edge of the 10 µm PMMA particles assembled on a fluorocarbon-coated Au-coated substrate. The corresponding average shape factor ($\vartheta_{\text{norm,av}}$) is also included.

5 $F(D)$-spectroscopy

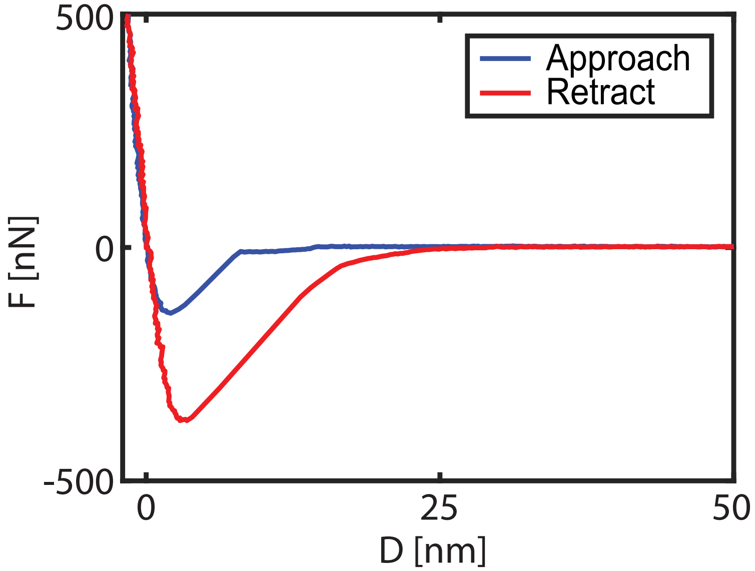

Figure S6: Force-distance ($F(D)$) spectroscopy curves (approach (blue) and retraction (red) of a diamond tip on a ITO substrate.

Force-spectroscopy spectroscopy was performed to determine the Young's modulus (Y). Force-distance curves ($F(D)$) are obtained by approaching and retracting a diamond tip towards and from the surface. A diamond probe is selected to avoid any deformation in the tip. Prior to the measurements, the deflection sensitivity of the cantilever is calibrated on a sapphire surface. Afterwards, the spring constant is determined using the thermal oscillation

method. An example of a $F(D)$ curve is shown in Fig S6. First, the tip is out of contact and no force is exerted on the probe (right part of the blue line in Fig. S6). When approaching, the probe jumps into contact with the substrate because the force gradient (the van der Waals and capillary force) is larger than the effective elastic constant of the cantilever. When the tip is in contact with the surface, the tip is pushed onto the surface till the load force (F_{L}) is reached.[3,4] The load force remains constant through all the experiments. From this point, the approach procedure is finished. During the retraction cycle, the tip is gently lifted from the surface. During the initial stage ($D < 0$ in Fig. S6), the deformation of the tip and sample is measured. In this case, a diamond tip is used and deformation of the tip can be excluded. In section S6, the extraction of the Young's modulus is discussed.

Further during the retraction phase ($D > 0$), the tip experiences different adhesion-type forces which are pulling on the cantilever. This is a combination of the van der Waals (F_{vdW}), the contact mechanics (F_{cm}) and the capillary (F_{cap}) force. The probe is lifted till it snap-out of contact ($D \approx 5$). From this moment, the probe is no longer in contact with the surface, but long-range forces still act on the probe (such as the electrostatic force, F_{e}).[4] When the probe is retracted even further away from the surface, the force acting on the probe is reaching zero and the procedure is over.

6 Determining the Young's modulus

For negative distances (D), the tip is contact with the surface. The tip is pushed onto the surface till the load force ($\mathrm{F_L}$) is reached.[3,4] The load force remains constant through all the experiments. From the linear regime of the curve (see inset of Fig. S7, $D < 0$), the Young's modulus is determined.

The calculation of the Young's modulus is based on the Hertz model with Sneddon's modification.[5] The model relates the applied loading force F to the indentation depth (δ), which is equal to the negative distance (D) part in Fig. S7. The relation is given for a

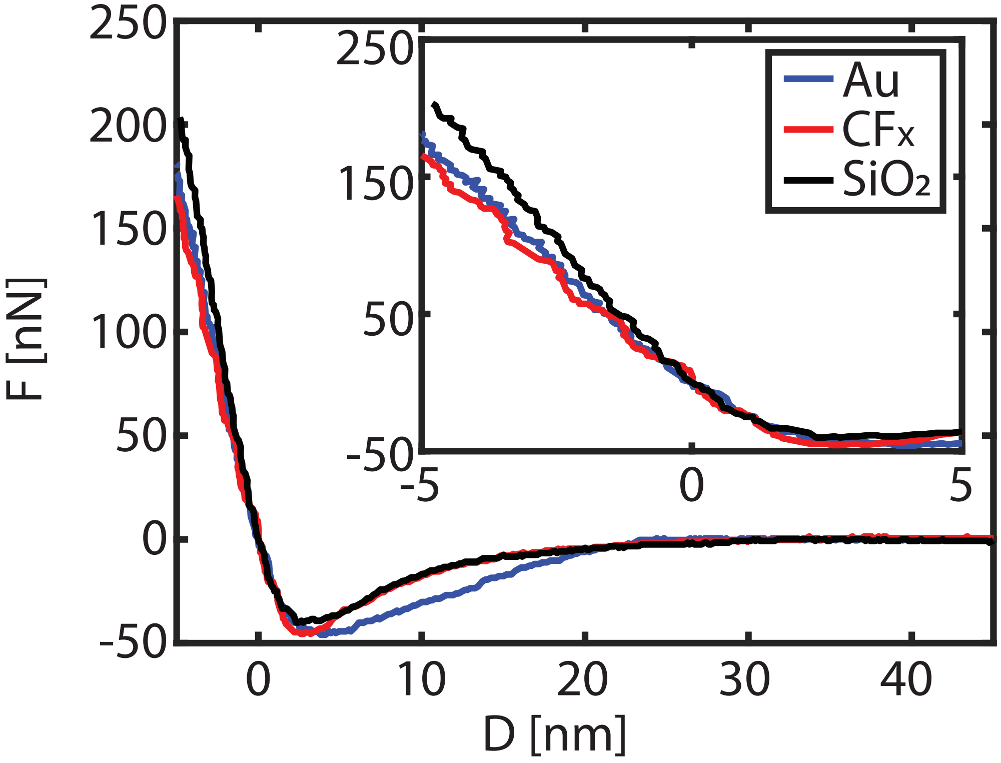

Figure S7: Force-distance ($F(D)$) spectroscopy curves of a diamond tip on a Au (blue), CF_x (red) and SiO_2 (black) substrate, respectively. Inset: zoomed graph on the indentation part of the $F(D)$ curve from which the Young's modulus is determined.

cone-shaped tip by

$$F_{\text{cone}} = \frac{2}{\pi}\frac{Y}{1-\nu^2}\tan\alpha\delta^2 \tag{S2}$$

with ν the Poisson ratio (0.3 used here) and α the half opening angle of the tip (22.5°). When equation S2 is rewritten, the Young's modulus is given by

$$Y_{\text{cone}} = \frac{\pi}{2}\frac{\Delta F_{\text{cone}}}{\Delta\delta^2}\frac{1-\nu^2}{\tan\alpha} \tag{S3}$$

The obtained values for the Young's modulus are depicted in Table S2. Do note that the obtained absolute values depend on the exact value of α, which is given by the supplier but can still deviate.

Table S1: The obtained values for the Young's modulus (Y) using the $F(D)$-spectroscopy depicted in Figure S7. The Young's modulus is calculated using equation S3.

	Y [GPa]
Au	45 ± 5
CF_x	21 ± 5
ITO	54 ± 5
SiO_2	89 ± 5
500 nm SiO_2	62 ± 5
8 μm SiO_2	34 ± 5

The Youngs moduli of the stamps are determined using the data in Figure 3b. The obtained data is measured using a colloidal probe, changing the geometry of the system. Therefore, a different model is used to extract the Youngs modulus.[5]

$$F_{\text{sphere}} = \frac{4}{3}\frac{Y}{1-\nu^2}\sqrt{R}\delta^{3/2} \tag{S4}$$

with R the radius of the colloid. When equation S4 is rewritten, the Young's modulus is given by

$$Y_{\text{sphere}} = \frac{3}{4}\frac{\Delta F_{\text{sphere}}}{\Delta\delta^{3/2}}\frac{1-\nu^2}{\sqrt{R}} \tag{S5}$$

Table S2: The obtained values for the Young's modulus (Y) using the $F(D)$-spectroscopy in Figure 3b of the main text. The Young's modulus is obtained using equation S5

	Y [MPa]
PDMS	2 ± 1
PEBA/α-FeOOH	30 ± 3
Al_{foil}	20000 ± 3000

7 Kelvin Probe Force Microscopy measurements

The Kelvin probe force microscopy (KPFM) experiments were conducted in a Bruker Icon atomic force microscopy (AFM) at ambient conditions with RH = 50 – 60 % (measured with TFA®Digital Professional Thermo-Hygrometer KLIMA BEE). A heavily doped n-type Si-cantilever with a resonance frequency of 65 kHz and a force constant of 0.6 N/m (HQ:NSC36, micromash) was used. The FM-KPFM mode is used, where the electrostatic force gradient is detected by the frequency shift of the cantilever oscillation. The tip was grounded in the KPFM-measurement, and therefore, the contact potential difference (V_{CPD}) is determined using:

$$V_{\text{CPD}} = \frac{\phi_{\text{s}} - \phi_{\text{tip}}}{\mid e \mid} \tag{S6}$$

with e the elementary charge and ϕ_{s} and ϕ_{tip} the work function of the sample and tip, respectively. From this equation, it is derived that a positive (negative) shift in V_{CPD} is a negatively (positively) charged surface.[6]

8 Monolayers on different stamps

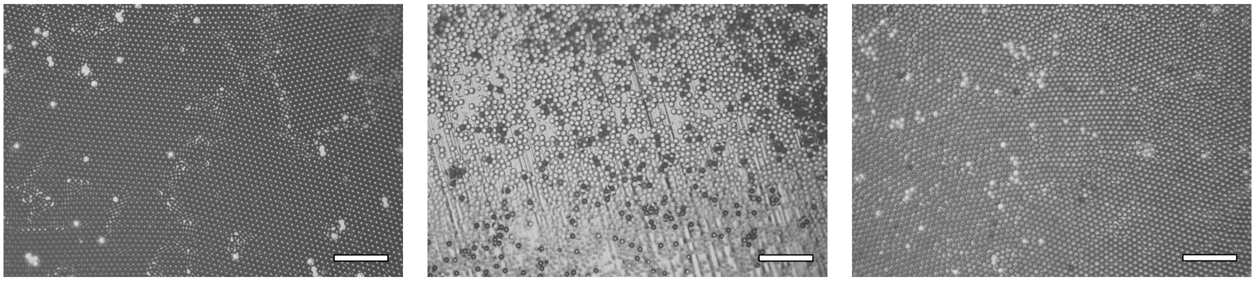

(a) (b) (c)

Figure S8: Microscope images of the three stamps; in (a) PMMA particles on the PDMS stamp, in (b) PS particles on the Aluminium stamp and in (c) silica particles on the PEBA/α-FeOOH stamp. The scalebar is 100 µm.

9 Wafer-scale assembly of tunable HCP crystal patterns

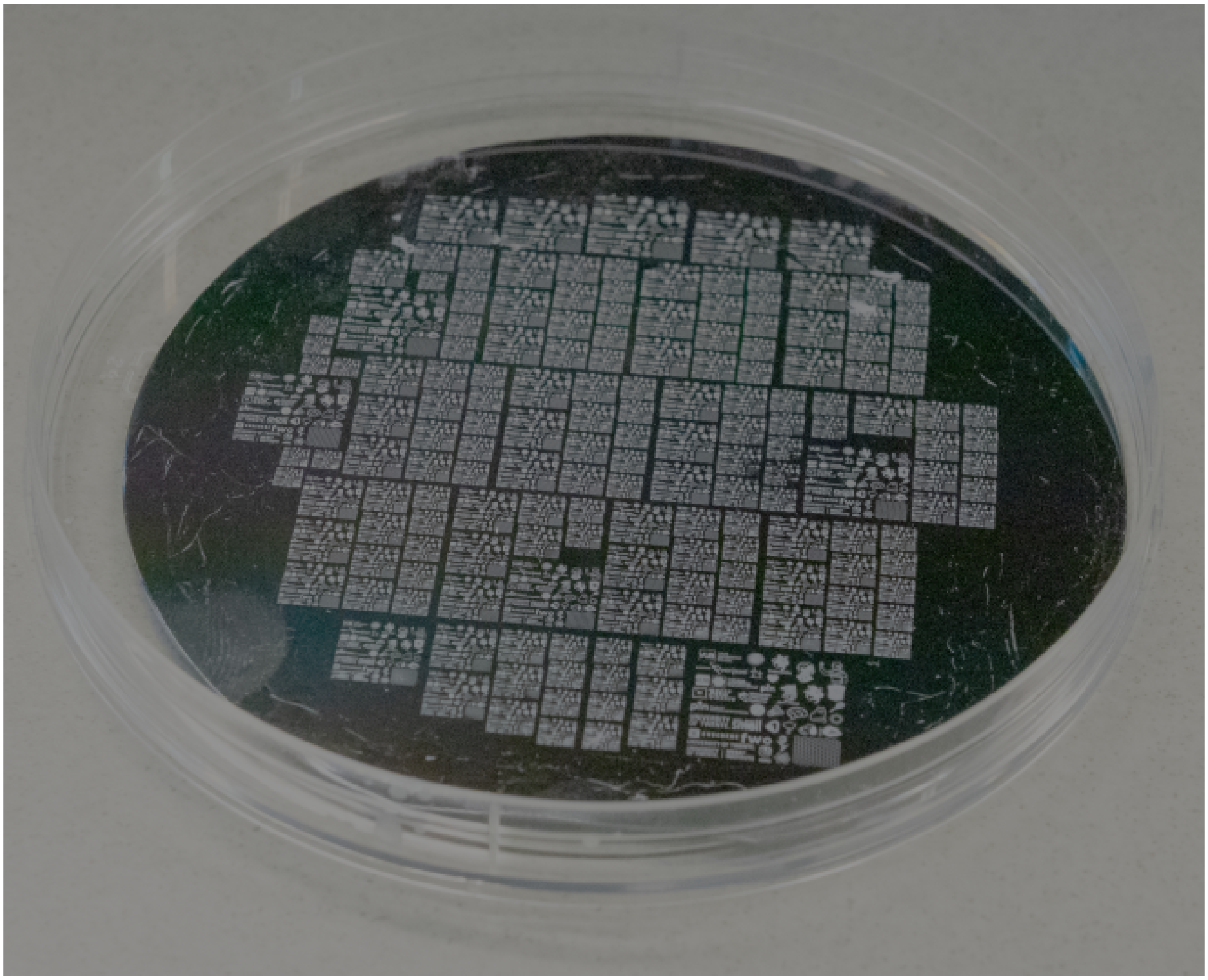

Figure S9: A 4-in. 8 μ SiO_2 wafer carrying arbitrary-fluorocarbon patches covered with 3 µm PMMA microspheres.

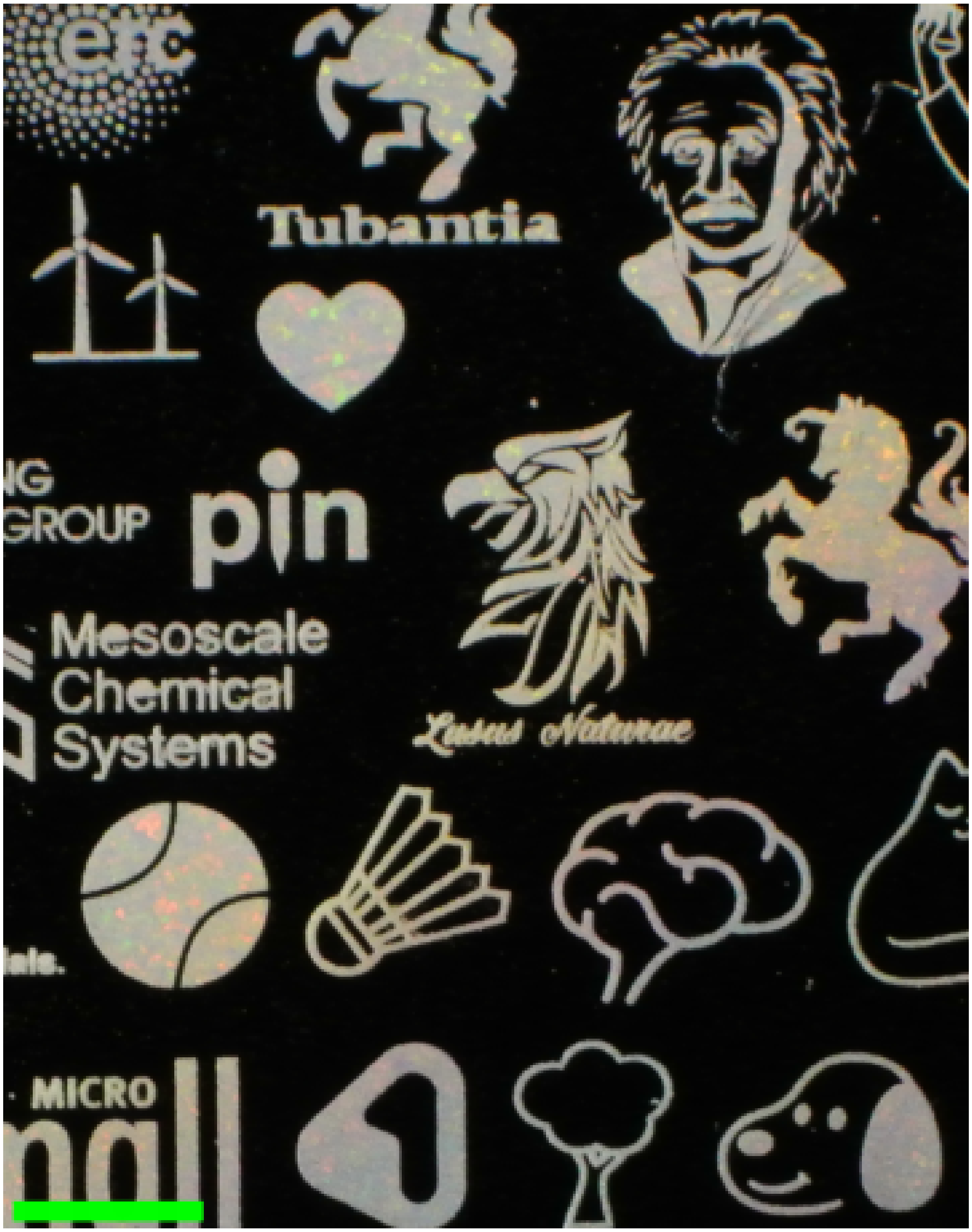

Figure S10: Iridescence structure colors were observed under the illumination of the fluorocarbon-patterned 8 µm SiO_2 wafer covered with 3 µm PMMA microspheres. Scale bar: 1 mm.

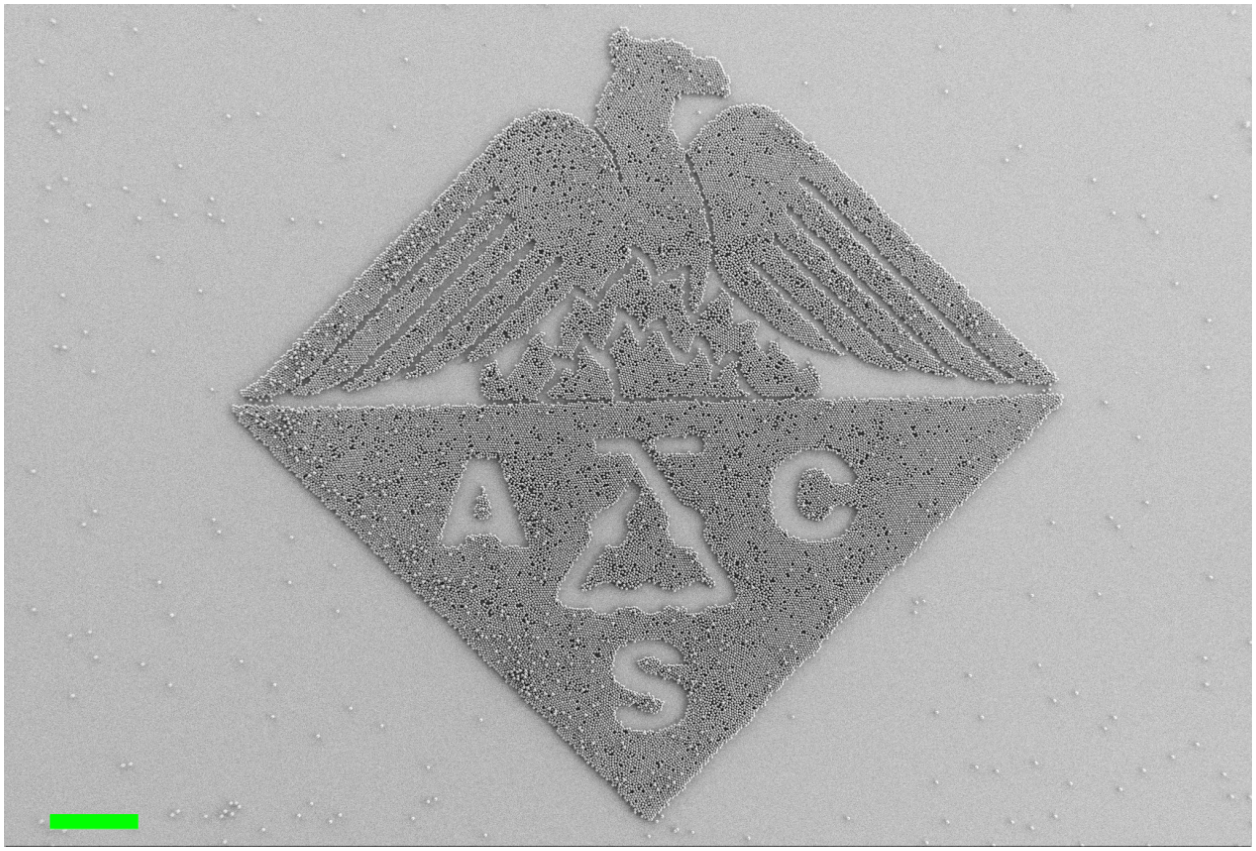

Figure S11: SEM image of 3 µm PMMA particles assembled on the a fluorocarbon-patterned structure resembling the ACS logo on an 8 µm SiO_2 wafer. Scale bar: 100 µm.

References

(1) Lotito, V.; Zambelli, T. Pattern formation in binary colloidal assemblies: Hidden symmetries in a kaleidoscope of structures. *Langmuir* **2018**, *34*, 7827–7843.

(2) Lotito, V.; Zambelli, T. Pattern detection in colloidal assembly: A mosaic of analysis techniques. *Advances in colloid and interface science* **2020**, *284*, 102252.

(3) Sotthewes, K.; Bampoulis, P.; Zandvliet, H. J. W.; Lohse, D.; Poelsema, B. Pressure-Induced Melting of Confined Ice. *ACS Nano* **2017**, *11*, 12723–12731, PMID: 29112376.

(4) Jimidar, I. S.; Kwiecinski, W.; Roozendaal, G.; Kooij, E. S.; Gardeniers, H. J.; Desmet, G.; Sotthewes, K. Influence of Wettability and Geometry on Contact Electrification between Nonionic Insulators. *ACS applied materials & interfaces* **2023**,

(5) Carl, P.; Schillers, H. Elasticity measurement of living cells with an atomic force microscope: data acquisition and processing. *Eur J Physiol* **2008**, *457*, 551–559.

(6) Melitz, W.; Shen, J.; Kummel, A. C.; Lee, S. Kelvin probe force microscopy and its application. *Surf. Sci. Rep.* **2011**, *66*, 1–27.
